# The Study of Biomechanics and Clinical Anatomy on a Novel Plate Designed for Posterolateral Tibial Plateau Fractures *via* Anterolateral Approach

**DOI:** 10.3389/fbioe.2022.818610

**Published:** 2022-03-08

**Authors:** Weizhi Ren, Wen Zhang, Shijie Jiang, Jian Peng, Chang She, Liubing Li, Yongtao Mao, Haibin Zhou, Wei Xu

**Affiliations:** ^1^ Department of Orthopedics, The Second Affiliated Hospital of Soochow University, Suzhou, China; ^2^ Orthopedic Institute, Soochow University, Suzhou, China; ^3^ Department of Orthopedics, Changzhou No. 2 People’s Hospital, The Affiliated Hospital of Nanjing Medical University, Changzhou, China

**Keywords:** biomechanical study, finite element analysis, clinical anatomy, internal fixation, tibial plateau fracture

## Abstract

There is no consensus about the optimal internal fixation selection for treatment of posterolateral tibial plateau fracture. This study described a novel plate through an anterolateral approach for posterolateral tibial plateau fractures (PTPFs). We evaluated the biomechanical performance of a novel plate and two conventional internal implants and investigated the anatomic feasibility of the novel plate. The fracture models were randomly assigned into six groups: Groups A–C were the model groups of posterolateral split fracture, fixed with the posterior buttress plate, the lateral locking plate, and the novel plate, respectively. Groups D–E were the model groups of posterolateral depression fracture, fixed with the posterior buttress plate, the lateral locking plate, and the novel plate, respectively. We evaluated the biomechanical performance of six model groups by the biomechanical testing and finite element analysis. Progressively increasing axial compressive loads were applied to each synthetic fracture model by using a customized indentor under 250–750 N loads. Meanwhile, we dissected 12 fresh frozen knee specimens and fixed them with the novel plate through the anterolateral approach. We recorded the adjacency of the novel plate to important anatomic structures. Biomechanical testing showed that the novel plate had the least displacement, followed by the posterior buttress plate, and the lateral plate had the most displacement in posterolateral split fracture. There was no significant difference in the displacement between the novel plate and the lateral plate at different loads in posterolateral depression fractures. And the posterior buttress plate showed the most displacement. In the finite element analysis, the maximum stress values of Groups A, B, and C were 383.76, 414.63, and 305.07 MPa under the load of 750 N, respectively. The maximum stress values of Groups D, E, and F were 474.28, 436.31, and 413.4 MPa under the load of 750 N, respectively. In the anatomic study, the placement of the novel plate had a low risk of damage to the important anatomic structures of knee posterolateral corner. The novel plate could be a great choice for the treatment of PTPFs due to better biomechanical performance and easy manipulation.

## 1 Introduction

Tibial plateau fractures are among the most common fractures in knee trauma. The fractures include a wide variety of fracture patterns, and four different quadrants of the tibial plateau may be involved (Xie et al., 2020). Among them, the treatment of posterolateral tibial plateau fracture (PTPF) has been always a challenging problem for orthopedic surgeons (LaPrade et al., 2003; Sassoon et al., 2014; Cho et al., 2017; Hoekstra et al., 2017). It’s difficult to identify the PTPF using the antero-posterior radiograph when the posterolateral (PL) fracture line is parallel to the coronal plane (Higgins et al., 2009; Kfuri and Schatzker, 2018). Thus, the PTPF was considered an uncommon fracture in the past, accounting for only 7%–10% of tibial plateau fractures (Connolly, 2005; Solomon et al., 2010; Chen et al., 2014). However, with the widespread use of computed tomography (CT) in recent years, the diagnosis rate of PTPF is higher than previously thought (Wang et al., 2017). Several studies have reported that PTPF accounts for approximately 15% of all tibial plateau fractures (Xiang et al., 2013), and approximately 54.3% of lateral tibial plateau fractures involve the PL column (Yi et al., 2020).

The PTPFs are the result of axial compressive forces combined with valgus stress with the knee in flexion (Xie et al., 2020), which most commonly causes depression fractures because of the convexity of the lateral tibial plateau (Sun et al., 2014). Besides depression fractures, PL column split fracture is a common type (Sun et al., 2014). The PL region of the knee plays a vital role in the flexion stabilization of the knee (Yu et al., 2012). As an intra-articular fracture, tibial plateau fracture requires anatomical reduction and rigid internal fixation. However, the operative treatment of PTPF is complex because of the special anatomical structures of the PL corner of the knee joint, including the fibular head, the fibular collateral ligament, the popliteus tendon, and the peroneal nerve, which impedes the exposure and fixation of the fracture fragments (Heidari et al., 2013; Giordano et al., 2020; Song et al., 2020). Currently, there is no uniform standard for selecting optimal internal fixation to treat PTPFs. The posterior buttress plate through various posterior approaches (He et al., 2013; Berber et al., 2014; Hoekstra et al., 2015; Gavaskar et al., 2016; Liu et al., 2016; Zhang et al., 2016) and the lateral locking plate through the anterolateral or lateral approaches (Hu et al., 2016; Kfuri et al., 2017) are still commonly used fixation methods in clinical practices. Serval results of biomechanical testing had shown that the posterior buttress plate could provide adequate stability in controlling the vertical displacement of PL fragment compared to the lateral locking plate (Zhang et al., 2012; Sun et al., 2018; Hu et al., 2020; Zhang et al., 2020). When the knee is flexed, there is a posterior and distal displacement of the PL fragment. And the posterior buttress could provide strong support for the shear fragment (Chang et al., 2009; Luo et al., 2010). However, the posterior approach could cause iatrogenic injury to the normal structure of the PL knee joint (Heidari et al., 2013; Solomon et al., 2013; Sun et al., 2017). Compared with the posterior approach, the anterolateral approach is a mature surgical method with little risk of injury to important anatomical structures. Some surgeons had successfully used the lateral locking plate to fix PTPFs by a posteriorly positioned through anterolateral or lateral approaches (Sun et al., 2017). However, whether a lateral locking compression plate could provide sufficient stability to the PTPF is still controversial (Sun et al., 2018). The proximal screws of the lateral locking plate are parallel to the coronal fracture line, which is a disadvantage (Cho et al., 2017). And failure cases of PTPF treated by the lateral locking plate were often encountered (Wang et al., 2017).

**FIGURE 1:**
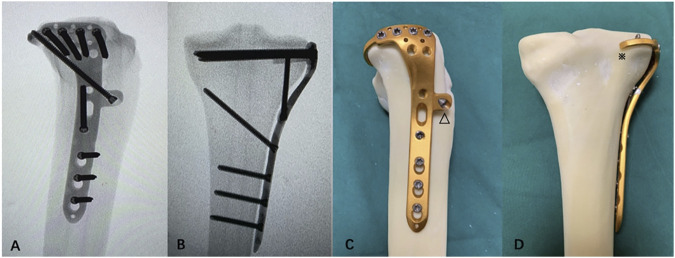
Mock-ups of the novel plate in intact synbones. **(A)** Lateral view of the X-ray image. **(B)** Anteroposterior view of the X-ray image. **(C)** Lateral view of the novel plate. **(D)** Anteroposterior view of the novel plate. The (△) anterior wing and (※) “hoop hook.”

The fixation options available for PTPFs are relatively single, with distal radial fixation plates or reconstruction plates being used via a posterior approach or a lateral locking compression plate through the anterolateral approach. Sometimes, the safety of the surgical approach and stability of the internal fixation cannot be met simultaneously. Thus, we developed a novel plate (Figure 1) through an anterolateral approach for PTPFs and applied for an invention patent in China (Patent No. ZL202010878681.7).

This study compared the biomechanical performance of this novel plate with other that of two conventional internal implants in the fixation of split fracture and depression fracture. Moreover, we performed an anatomical study to investigate the feasibility of clinical application for the novel plate.

## 2 Materials and Methods

### 2.1 Fracture Models Construction and Fixation

In this study, 48 right synthetic tibias (type 1110; Sybone AG, Swiss) were used to make models of PL tibial plateau fracture with reference to previous studies (Zhang et al., 2012; Sun et al., 2018). The synthetic tibia model was made of a rigid foam cortical shell, which was filled with cancellous material, which was purchased from a single manufacturing batch to ensure the same material property, architecture, and geometry.

#### 2.1.1 Fracture Models of Posterolateral Split Tibial Plateau Fracture

**FIGURE 2:**
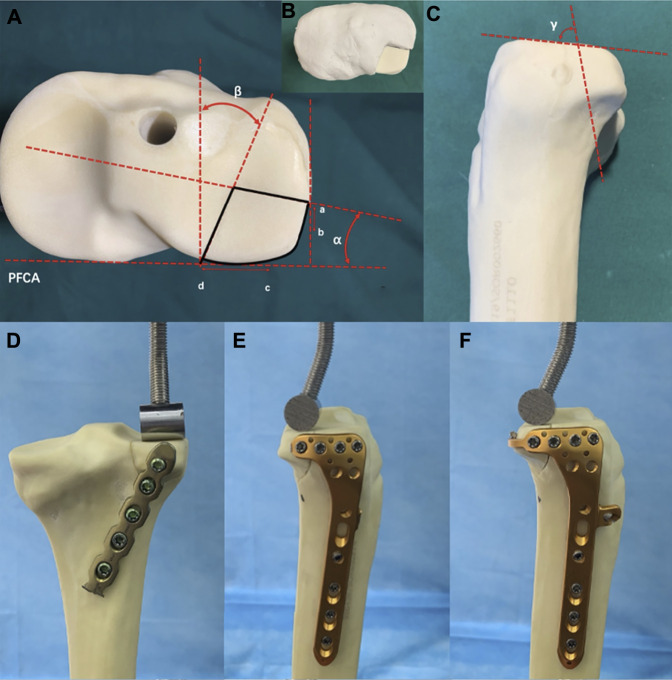
Cranial and lateral views of the posterolateral (PL) split fracture model of tibial plateau and biomechanical test. **(A)** Cranial view. PFCA, posterior femoral condyle axis; point a, lateral exit point of the PL fracture; point b, anterior edge of the articular facet of fibular head; point c, medial edge of the articular facet of fibular head; point d, posterior exit point of the PL fracture; angleα, angle between the lateral fracture line of the PL fragment and the PFCA; angleβ, angle between the medial l fracture line of the PL fragment and the line perpendicular to the PFCA. **(B)** Model made of plastic clay. **(C)** Lateral view. Angle γ, angle between the joint line of the PL fragment with the coronal fracture line [Values of models based on data from the morphological measurements by Sohn et al. (2015)]. **(D)** Fixation of the posterior buttress plate in PL split fracture. **(E)** Fixation of lateral locking plate in the PL split fracture. **(F)** Fixation of novel plate in the PL split fracture.

On the basis of the data from the morphological measurements by Sohn et al. (2015) and published literature (Sun et al., 2018), the PL part of the synthetic tibiae was sawed to simulate a PL split fracture. Figures 2A–C demonstrate the modeling of a PL split fracture. A thin blade saw was used to perform the osteotomy. Geometrical measurements were measured by Auto CAD software (Auto CAD, 2020; Autodesk, San Rafael, CA, United States). And we made a custom clay mold to assist us in taking the measurements of the PL fragment (Figure 2B). Moreover, all geometric measurements and preparations were performed by a single surgeon.

#### 2.1.2 Fracture Models of Posterolateral Depression Tibial Plateau Fracture

**FIGURE 3:**
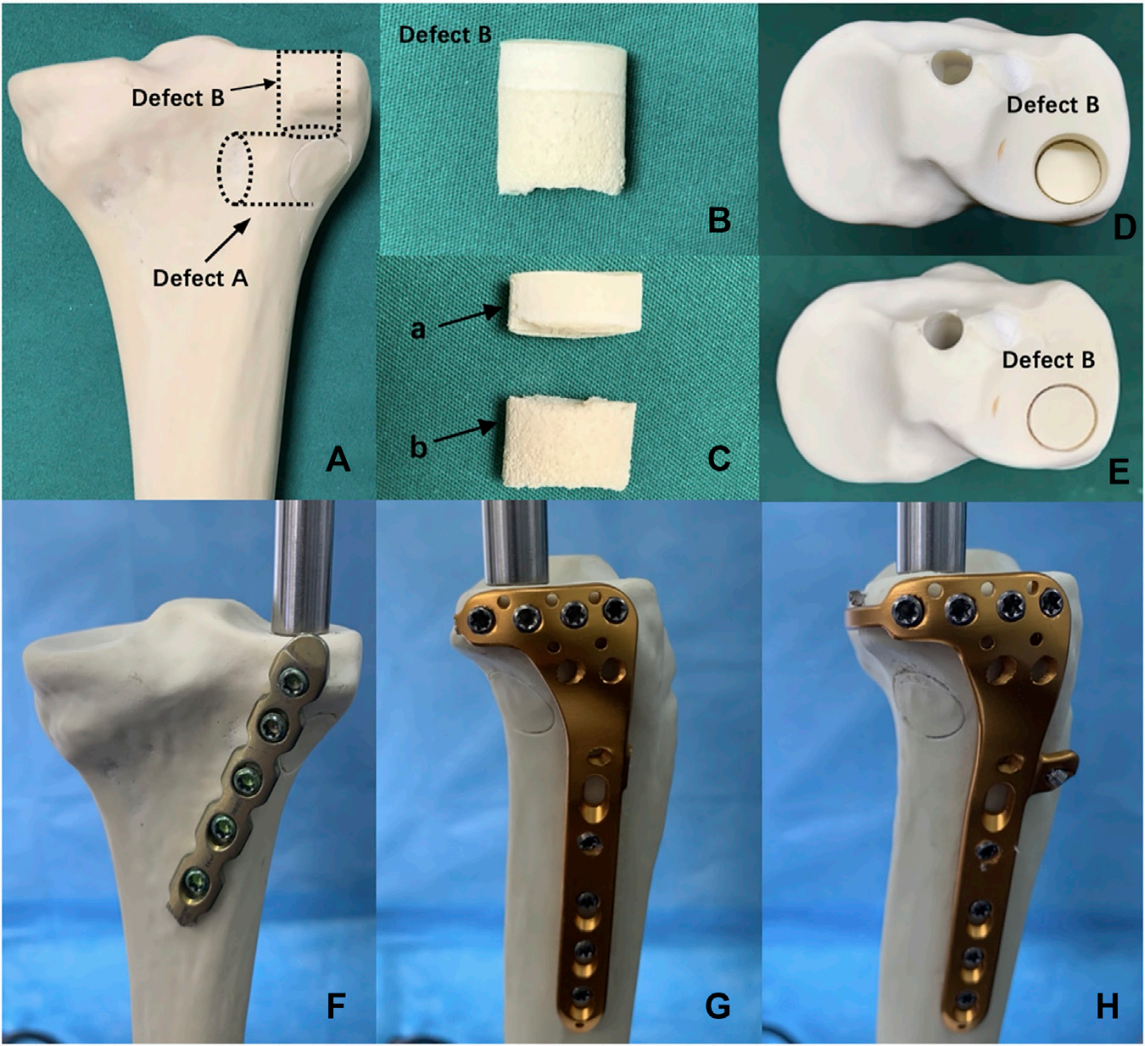
Posterolateral (PL) depression fracture model of tibial plateau and biomechanical test. **(A)** Defect A—15-mm-diameter cylindrical defect in the lateral tibial condyle created by a hollow drill-bit. Defect B—14-mm-diameter cylindrical defect in the PL tibial plateau created by a hollow drill-bit. **(B)** Defect B was put out from tibial plateau. **(C)** Defect B was divided into the two parts, (a) proximal articular surface and (b) distal cancellous bone. **(D)** PL depression fracture model. **(E)** PL depression fracture model was reduced anatomically. **(F)** Fixation of posterior buttress plate in the PL depression fracture. **(G)** Fixation of lateral locking compression plate in the PL depression fracture. **(H)** Fixation of the novel plate in the PL depression fracture.

Fracture models that simulate the depression fractures of PL tibial plateau were created in synthetic tibial specimens. Figures 3A–E demonstrate the modeling of a PL depression fracture, referring to the lateral tibial plateau fracture model by previous studies (Welch et al., 2003; Jordan et al., 2016). A 14-mm-diameter depression fragment on the PL tibial plateau was created similarly to the dimension of the PL split fragment. To avoid the adverse effects of an irregular shape of the PL fracture fragment, we used a thin hollow drill to ensure that the shape of the fragment was uniform and regular. First, a cylindrical defect that is 15 mm in diameter (Defect A) was created in the PL tibial condyle far from 15 mm below the tibial plateau surface by using a hollow drill-bit. Second, by using a 14 mm drill bit, a cylindrical defect that is 14 mm in diameter (Defect B) was created within the PL tibial plateau. Defect A and B crossed mutually, and Defect B could be taken out easily. Defect B was divided into two parts, the distal cancellous bone simulating the depression part, and the proximal cortical bone simulating the articular cartilage and subchondral bone. Finally, the removed cancellous and cortical bones were backfilled in sequence to restore anatomical morphology. All geometric measurements and preparations were taken by a single surgeon.

#### 2.1.3 Fracture Fixation Groups

**FIGURE 4:**
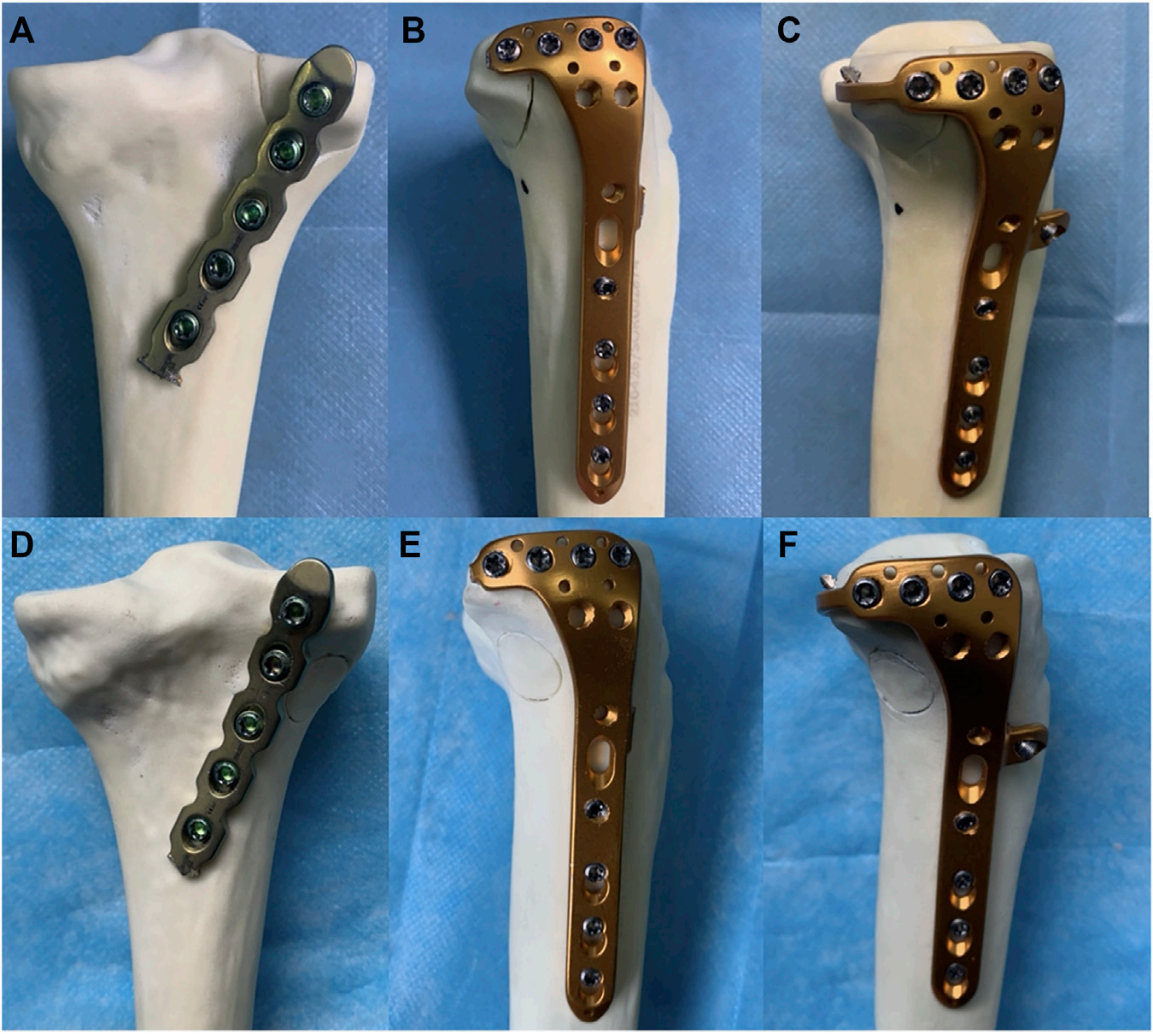
Six different internal fixation models of the posterolateral (PL) fracture. **(A)** The posterior buttress plate fixation in PL split fracture. **(B)** The lateral tibia locking compression plate in PL split fracture. **(C)** The novel plate in PL split fracture. **(D)** The posterior buttress plate fixation in PL depression fracture. **(E)** The lateral tibia locking compression plate in PL depression fracture. **(F)** The novel plate in PL depression fracture.

Forty-eight synthetic tibias were randomly assigned to six groups (A–F, eight per group). Groups A to C were the model groups of PL split tibial plateau fractures. Groups D–F were the model groups of PL depression tibial plateau fractures (Figure 4). Group A: A posterior five-hole buttress plate (straight, 3.5 mm) was used for the fixation of the PL split fracture. The posterior buttress plate was contoured and implanted from the proximal lateral aspect of the posterior tibia to the distal medial aspect of the tibia. Group B: A lateral locking plate (L-shaped, 3.5 mm) was used for the PL split fracture model, and the transverse arm of the L-shaped plate had four holes. According to previous studies (Hu et al., 2016; Sun et al., 2018), the lateral locking plate was placed as posteriorly as possible to fix the PL fragment with one or at most two screws. Group C: The novel plate (L-shaped, 3.5 mm) was used for PL split fracture model. The novel plate was placed as posteriorly as possible and at the same position as the lateral locking plate. Compared with Group B, the addition of the anterior wing screw and the “hoop hook” of the novel plate fixed the PL split fragment together. Group D: The posterior buttress plate was used for the PL depression fracture model. Group E: A lateral locking plate was used for the PL depression fracture. The lateral locking plate was placed as posteriorly as possible to fix the PL fragment with two screws. Group F: The novel plate was used for the PL depression fracture. The novel plate was also placed as posteriorly as possible and at the same position as the lateral locking plate.

The same manufacturer made the implants to ensure material and design consistency. All fracture models were reduced and fixed by a single orthopedic surgeon.

### 2.2 Biomechanical Testing

**FIGURE 5:**
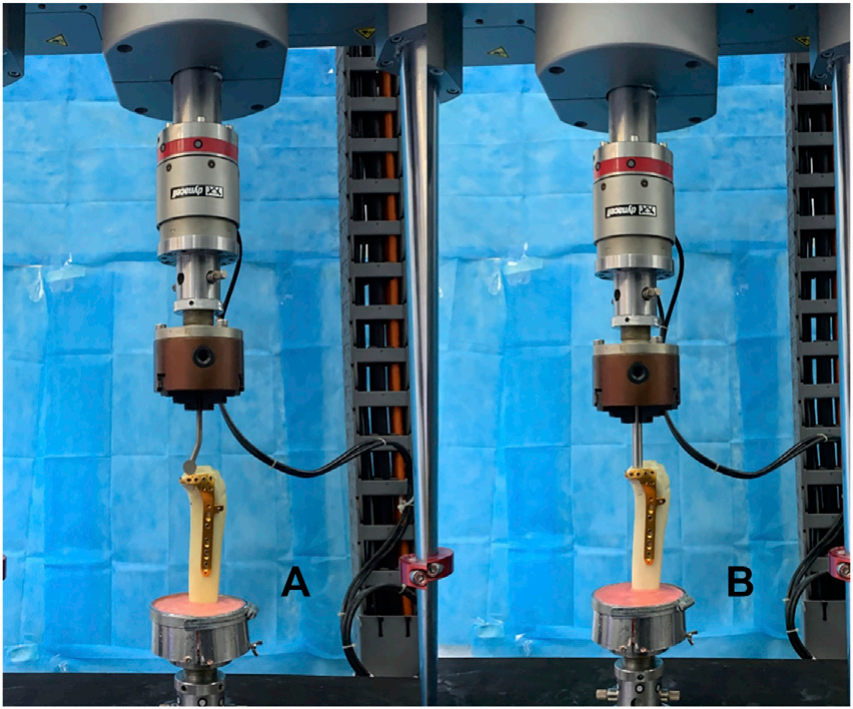
Positioning of synthetic tibia model within the machine. **(A)** Fixation of the novel plate in the posterolateral (PL) split fracture. **(B)** Fixation of the novel plate in the PL depression fracture.

Each potted synthetic tibia was placed vertically in a material-testing machine (In-stronE10000, Instron Corporation Norwood, MA, United States) (Figure 5). The load was applied to the PL split fragment through a custom T-shaped applicator. The applicator was bent at an angle of 17° so that the angle was parallel to the fracture line in the sagittal plane to simulate the shearing force of the lateral femoral condyle (Feng et al., 2021) (Figures 2D–F). Usually, the PL tibial plateau depression fracture was mainly affected by axial forces during normal gait (Jordan et al., 2016). Therefore, for PL depression fracture, an axial load was applied with a custom cylindrical indentor on the PL depression fragment (Figures 3F–H). The diameter of the cylinder was slightly smaller than the diameter of the fragment. And the indentor was fixed in advance as the reference location to ensure the models were in the same location.

Biomechanical loading on the knee joint during normal gait is approximately two to three times body weight (Taylor et al., 2004), and the loading ratio of the medial and lateral plateau was approximately 55% and 45%, respectively (Zhao et al., 2007). Therefore, when the human body weight (BW) was set at 60 kg, we chose three different axial peak loads of 250, 500, and 750 N (1–3 times BW) to simulate the loads on the lateral plateau during the state of a single-leg stance. The biomechanical testing was to simulate the static phase for different fixation methods in the material testing machine. After mounting each fracture model, progressively increased axial compressive loads were applied to each model with a load speed of 10 N/s. Axial displacement from the initial position to axial peak loads was continuously captured using Bluehill software (Instron, Norwood, MA, United States). Load–displacement curves were generated for each model. Moreover, failure form was defined as the situation when the vertical displacement of the PL fragment was 3 mm (Ali et al., 2002). And the maximum peak force was set at 750 N or the force at a displacement of 3 mm for PL fragment. Finally, the displacements at three load levels (250, 500, and 750 N) and failure load were chosen to evaluate the biomechanical stability of three different fixations.

### 2.3 Statistical Analysis

One-way analysis of variance was performed on the data to determine whether fragment displacement and final failure differed among these fixation models. Fisher’s post hoc test and least significant difference criterion were used to correct for multiple group comparisons. The level of significance was set to 0.05 for all statistical tests. All statistical analyses were computed using SPSS version 19.0 software (SPSS, Inc., Chicago, IL, United States).

### 2.4 Finite Element Analysis

**FIGURE 6:**
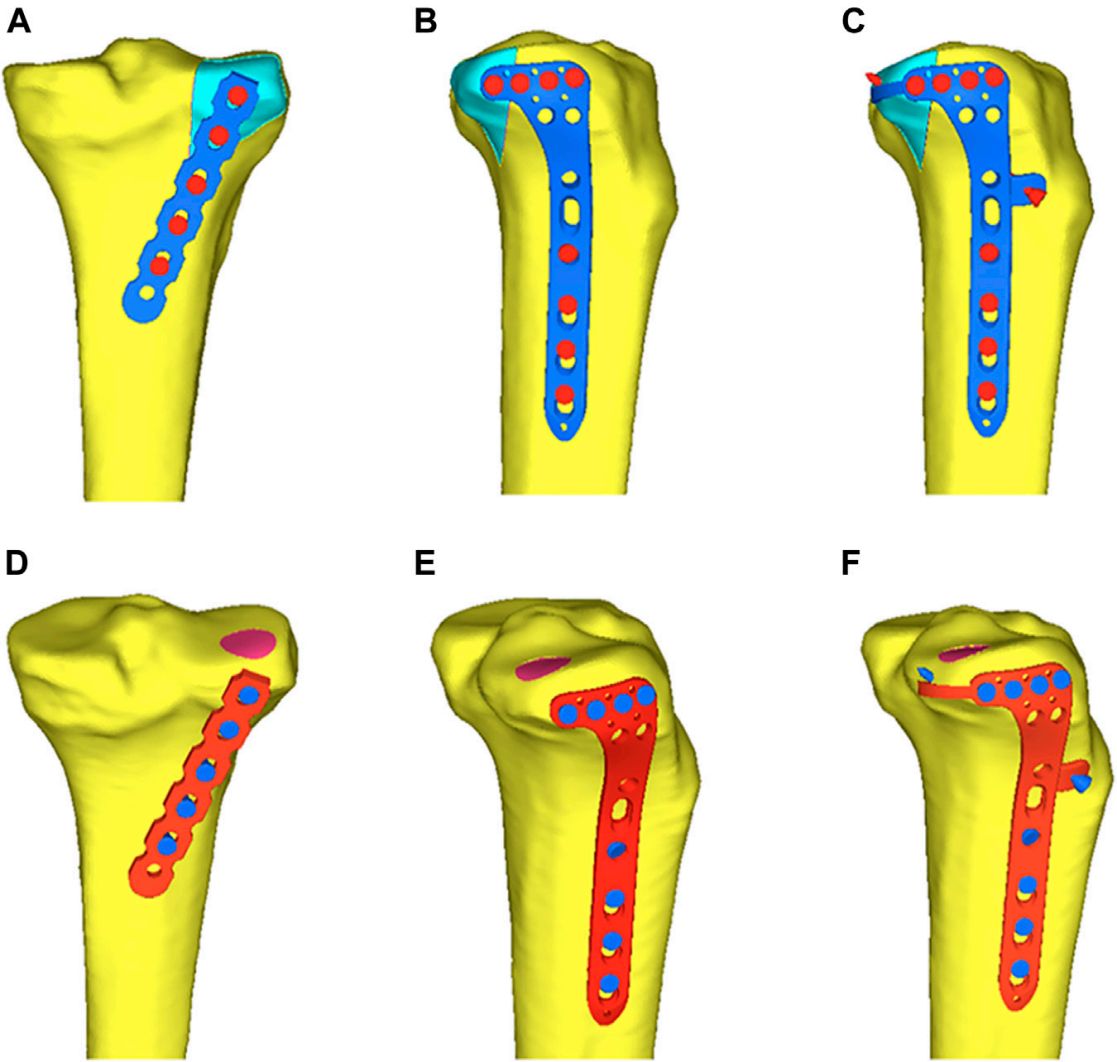
Six different internal fixation after assembly of the finite element model. **(A)** Fixation of the posterior buttress plate in posterolateral (PL) split fracture. **(B)** Fixation of the lateral locking plate in PL split fracture. **(C)** Fixation of the novel plate in the PL split fracture. **(D)** Fixation of the posterior buttress plate in PL depression fracture. **(E)** Fixation of the lateral locking plate in PL depression fracture. **(F)** Fixation of the novel plate in PL depression fracture.

A 30-year-old healthy male volunteer was recruited without a history of knee and systemic disease. By using a 64-row spiral CT scanner, a layer thickness of 0.625 mm CT scan was performed from the knee to the ankle. The CT image was stored in a DICOM format file into the medical three-dimensional reconstruction software Mimics (version 19.0, Materialise, Leuven, Belgium). A three-dimensional model of the tibia was built on the basis of the gray value of the tissue and segmentation of the region. This model was incorporated into software Geomagic-Studio (version 12, Geomagic, NC State, United States) for a smoothing process to correct the three-dimensional model surface. The different parts of finite element model were imported into software Hypermesh (version 2017, Altair, Inc., United States), a meshing tool for finite element analysis, and meshed using quadratic tetrahedral elements Solid187. The tibia was considered isotropic linear elastic and homogeneous. Each model consisted of quadratic tetrahedron elements from 0.5 to 1.0 mm in size. A convergence test was performed on all models to ensure the maximum change was less than 1%. Table 1 showed the material assignment. The Young’s modulus and Poisson’s ratio were obtained from the literature (Fan et al., 2008; Qiu et al., 2011; Anwar et al., 2017; Anwar et al., 2018; Huang et al., 2019). The three-dimensional model of the plate and screws was made according to the specifications of the manufacturer using computer-aided design software Creo Parametric (PTC, Inc., United States). All contact conditions between fracture fragments and the implant were defined as frictional contacts. We chose a friction coefficient of 0.4 (Rancourt et al., 1990; Viceconti et al., 2000). The tibia model was imported into software Geomagic Studio (3D system Inc., Rock Hill, SC, United States), and the fracture line was cut to develop the PL tibial plateau split fracture and the PL depression tibial plateau fracture (Figure 6). Internal fixations were assembled with fracture models to complete the internal fixation models of the PL tibial plateau fracture by using software Creo Parametric on the basis of the relative data. All contact Group A: A posterior buttress plate was used for the PL split fracture. Group B: A lateral locking compression plate was used for PL split fracture. Group C: The novel plate was used for the PL split fracture. Group D: A posterior buttress plate was used for the PL depression fracture. Group E: A lateral locking compression plate was used for the PL depression fracture. Group F: The novel plate was used for the PL depression fracture. Table 2 shows the numbers of elements and nodes of the various models in the experiment. The inferior of the distal tibia was fixed in all degrees of freedom. The PL split/depression fragments were compressed using three different loadings (250, 500, and 750 N) with the loading direction parallel to the *Z*-axis of the tibial plateau. All models were analyzed by software ANSYS Mechanical APDL 19.0 (ANSYS, Inc., United States). The finite element analysis was performed simulating a static test for different fixation methods. Moreover, the finite element model was validated with the published data, and the procedure was explained in our previous study (Zhou et al., 2021).

**TABLE 1:** Properties considered for the materials.

Material	Young’s modulus (MPa)	Poisson’s ratio
Cortical bone	14000	0.3
Cancellous bone	700	0.3
Plate	110000	0.3
Screw	110000	0.3

**TABLE 2:** Number of nodes and elements for the six models.

Model	Nodes	Elements
Group A	912809	565108
Group B	902541	552457
Group C	945150	591667
Group D	913706	577132
Group E	913248	572768
Group F	945862	591718

We analyzed the vertical displacement of the PL fragments, the von Mises stress distribution, and the maximum von Mises stress of each internal fixation under axial loads.

### 2.5 Anatomic Study

Twelve fresh frozen knee specimens were used in this study. None of the knee joints had signs of previous injury, abnormality, or disease. The mean age of the donors was 61.3 years (range: 46–72 years).

Each lower limb was dissected using the anterolateral approach. The incision was made, starting 1 cm proximal to the knee joint line along the midline of the lateral side, toward Gerdy’s tubercle, and then down to the lateral side of the tibial tuberosity. Thereafter, a fascial incision was made in the same way as the skin incision. The iliotibial band along the backside was retracted anteriorly and opened to separate the distal fiber bundles from Gerdy’s tubercle. Furthermore, the iliotibial band was performed a sharp dissection along the upper edge of the fibular head. The coronary ligament of the meniscus was cut open to visualize the PL tibial plateau. With the knee flexed at 60°, the PL articular surface could be easily visualized by the internal rotation and varus of the tibia. And the plate position could be adjusted to match adequate and optimal anatomic structures through the superior fibular head space. Moreover, the PL tibial plateau was dissected carefully to show the adjacency of the novel plate to important anatomic structures (the popliteus tendon, the common peroneal nerve, lateral inferior genicular artery, and popliteal arteriovenous vessels).

## 3 Results

### 3.1 Biomechanical Testing

#### 3.1.1 Posterolateral Split Tibial Plateau Fractures

Table 3 showed the vertical displacement of the PL fragment under three different axial loads. There was a displacement hierarchy of the fragment at different load levels in PL split fracture: the novel plate (Group C) had the least displacement, followed by the posterior buttress plate (Group A), and the lateral locking plate (Group B) had the most displacement. The differences among the three groups were statistically significant (*p* < 0.001).

**TABLE 3:** Vertical displacement of the posterolateral split fracture at three different load levels, load to failure.

Groups	Vertical displacement (mm)			Load to failure (N)
	250 N	500 N	750 N	
A	1.21 ± 0.08	2.17 ± 0.16	3.47 ± 0.40	605.42 ± 34.04
B	1.47 ± 0.10	3.26 ± 0.22	4.85 ± 0.73	431.32 ± 33.01
C	0.96 ± 0.04	1.74 ± 0.08	2.66 ± 0.17	776.71 ± 12.74
P (A-B)^a^	0.001	0.001	0.004	0.001
P (B-C)^a^	0.001	0.001	0.001	0.001
P (C-A)^a^	0.001	0.001	0.001	0.001

a Significant difference (Group A. The posterior buttress plate. Group B. The lateral tibia locking compression plate. Group C. The novel plate).

Table 3 showed the failure loads of each specimen. The failure load of Group C was the highest and was significantly higher than that of the other two implants (*p* < 0.001). And there was also a significant difference in the failure load between Groups A and B (*p* < 0.001). The posterior buttress plate (Group A) bore more load than the lateral locking plate (Group B). Failure load was 605.42 ± 34.04 N for the posterior buttress plate (Group A), 431.32 ± 33.01 N for the lateral locking plate (Group B), and 776.71 ± 12.74 N for the novel plate (Group C). This result showed that the novel plate had a better biomechanical advantage over the posterior buttress plate and lateral plate in terms of vertical displacement and failure load for PL split fracture.

#### 3.1.2 Posterolateral Depression Tibial Plateau Fractures

Table 4 showed the vertical displacement of the depression fragment under three different axial loads. The vertical displacements at 250, 500, and 750 N loads of the novel plate (Group F) and the lateral locking plate (Group E) were significantly smaller than those of the posterior buttress plate (Group D) (*p* < 0.05). Although there was no significantly difference between Groups F and E, there was a hierarchy of vertical displacement of the depression fragment: Group F had the least displacement, Group E had second, and Group D had the most displacement.

**TABLE 4:** Vertical displacement of the posterolateral depression fracture at three different load levels, load to failure.

Groups	Vertical displacement (mm)			Load to failure (N)
	250 N	500 N	750 N	
D	1.25 ± 0.08	2.13 ± 0.13	2.71 ± 0.14	846.30 ± 52.18
E	0.90 ± 0.09	1.45 ± 0.23	2.28 ± 0.21	1014.95 ± 70.87
F	0.87 ± 0.10	1.38 ± 0.14	2.11 ± 0.07	1034.79 ± 39.05
P (D-E)^a^	0.001	0.001	0.014	0.001
P (E-F)	0.588	0.399	0.236	0.677
P (F-D)^a^	0.001	0.001	0.003	0.006

a Significant difference (Group D. The posterior buttress plate. Group E. The lateral tibia locking compression plate. Group F. The novel plate).

Table 4 showed the failure loads of each specimen. Groups E and F had higher failure load, which was significantly higher than that of Group D (*p* < 0.05). The failure load was 846.30 ± 52.18 N for the posterior buttress plate (Group D), 1014.95 ± 70.87 N for the lateral locking plate (Group E), and 1034.79 ± 39.05 N for the novel plate (Group F). The novel plate provided the same biomechanical stability as the lateral locking plate for PL depression tibial plateau fracture, and both were superior to the posterior buttress plate.

### 3.2 Finite Element Analysis

#### 3.2.1 Posterolateral Split Tibial Plateau Fractures

**FIGURE 7:**
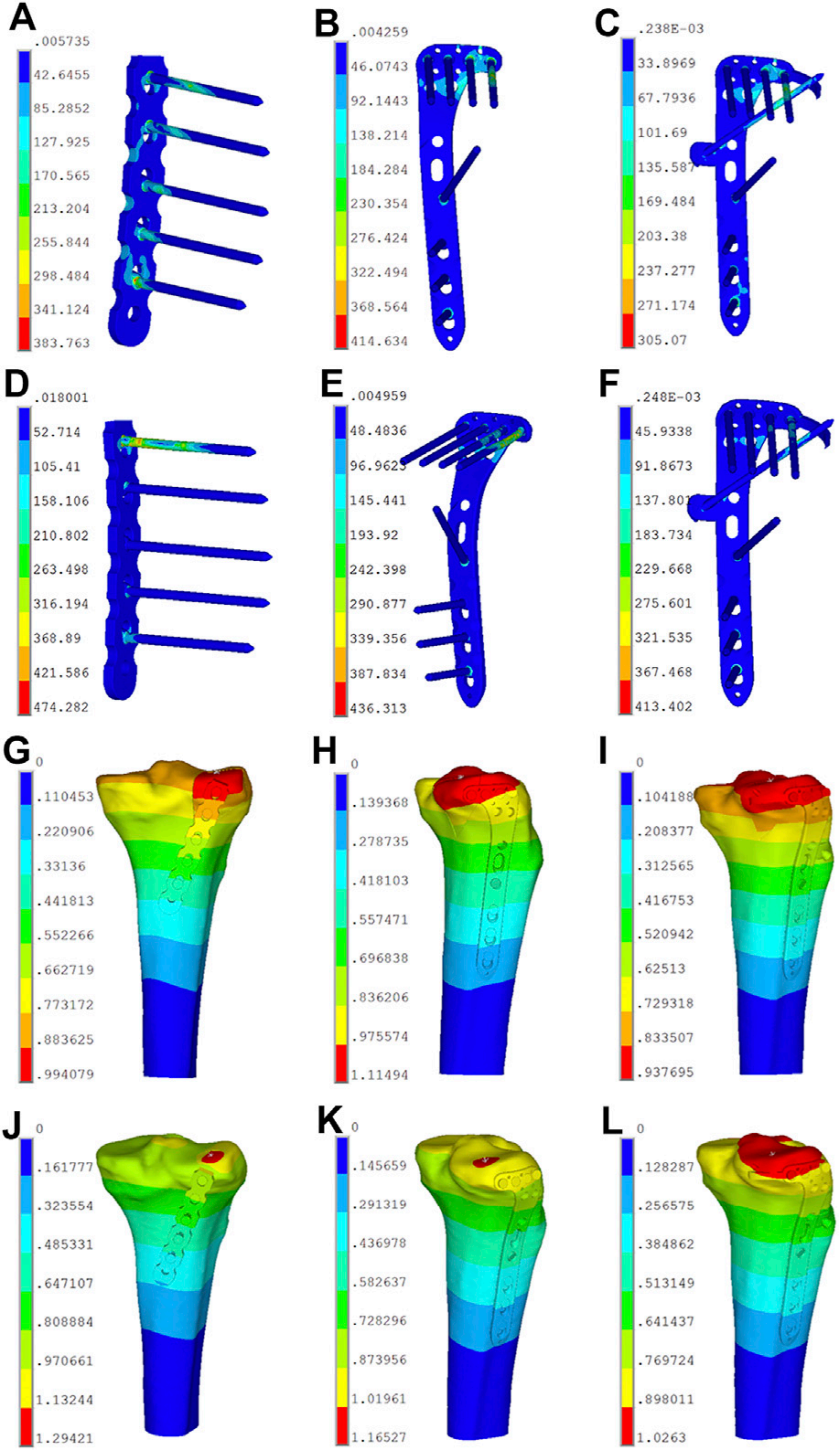
Stress distribution diagram and displacement field of the six finite element models. **(A)** Stress distribution of model A in posterolateral (PL) split fracture. **(B)** Stress distribution of model B in PL split fracture. **(C)** Stress distribution of model C in PL split fracture. **(D)** Stress distribution of model D in PL depression fracture. **(E)** Stress distribution of model E in PL depression fracture. **(F)** Stress distribution of model F in PL depression fracture. **(G)** Displacement field of model A in posterolateral (PL) split fracture. **(H)** Displacement field of model B in PL split fracture. **(I)** Displacement field of model C in PL split fracture. **(J)** Displacement field of model D in PL depression fracture. **(K)** Displacement field of model E in PL depression fracture. **(L)** Displacement field of model F in PL depression fracture.

When an axial load of 750 N was applied to three internal fixations, the maximum displacement in Groups A, B, and C were 0.99, 1.11, and 0.94 mm, respectively (Figures 7G–I). The displacement trends of the three different loads (250, 500, and 750 N) were consistent. Moreover, the displacements of the PL split fragment in each of the three groups gradually increased under loads from 250 to 750 N, and Table 5 showed the displacements of the different loads.

**TABLE 5:** Maximum displacement of the finite element models of posterolateral tibial plateau fracture.

Group	Max displacement (mm)		
	250 N	500 N	750 N
A	0.33	0.66	0.99
B	0.37	0.74	1.11
C	0.31	0.63	0.94
D	0.43	0.86	1.29
E	0.39	0.78	1.17
F	0.34	0.68	1.03

The von Mises stress distribution of the posterior buttress plate (Group A) focused on the two proximal screws in contact with the fracture line and the local area of the plate between the plate and screw (Figure 7A). The von Mises stress distribution of the lateral locking plate (Group B) focused on the corner junction of the transverse and longitudinal arms, and the screw was located most posteriorly (Figure 7B). The von Mises stress distribution of the novel plate body (Group C) was similar to that of the lateral plate. Moreover, the contact point of the “hoop hook” and the anterior wing screw obtained the stress concentration point (Figure 7C). The concentration point of the “hoop hook” was relatively low, thus suggesting that no mechanical damage would be expected in the novel plate. When the load increased, the von Mises stress increased in all three internal fixations. The maximum von Mises stress of Groups A, B, and C were 383.76, 414.63, and 305.07 MPa under an axial load of 750 N, respectively. The von Mises stress distribution was consistent with the increase of the axial loads from 250 to 500 N, and Table 6 showed the von Mises stress values of different loads. In addition, the maximum von Mises stress in bone of Groups A, B, and C were 76.70, 55.68, and 56.30 MPa under an axial load of 750 N, respectively (Table 7). The maximum von Mises stresses in bone by the novel plate and the lateral locking plate were decreased compared to the posterior buttress plate for PL split fracture.

**TABLE 6:** Maximum von Mises stress of the finite element models of posterolateral tibial plateau fracture in internal fixation method.

Group	Max von Mises stress (MPa)		
	250 N	500 N	750 N
A	127.92	255.84	383.76
B	138.21	276.42	414.63
C	101.69	203.38	305.07
D	158.09	316.29	474.28
E	145.44	290.88	436.31
F	137.80	275.60	413.40

**TABLE 7:** Maximum von Mises stress of the finite element models of posterolateral fracture in bone.

Group	Max von Mises stress (MPa)		
	250 N	500 N	750 N
A	25.57	51.14	76.70
B	18.56	37.12	55.68
C	18.77	37.53	56.30
D	47.97	95.94	143.92
E	24.24	48.49	72.73
F	22.43	44.86	67.29

#### 3.2.2 Posterolateral Depression Tibial Plateau Fractures

When axial loads of 250, 500, and 750 N were applied to three internal fixations, the displacement of the depression fracture fragments with the same load in the novel plate (Group F) was smaller than in the lateral locking plate (Group E) and the posterior buttress plate (Group D). The displacement values in Groups D, E, and F were 1.29, 1.17, and 1.02 mm under an axial load of 750 N, respectively (Figures 7J–L). The vertical displacements of the PL depression fragment in each group gradually increased under loads from 250 to 750 N, and Table 5 showed the displacement values of the different loads.

The von Mises stress distribution of the posterior buttress plate (Group D) mainly focused on the first proximal screw that is in contact with the fracture fragment and the local area of the plate between the plate and screw (Figure 7D). The von Mises stress distribution of the lateral locking plate (Group E) mainly focused on the corner junction of the transverse and longitudinal arms (Figure 7E). The von Mises stress distribution of the novel plate body (Group F) was also similar to that of the lateral locking plate (Figure 7F). However, the maximum von Mises stress of the corner junction of the novel plate body was smaller than that of the lateral locking plate. Moreover, the contact point of the “hoop hook” and the anterior wing screw also obtained the von Mises stress concentration point. The concentration point of the “hoop hook” was relatively low, suggesting that the risk of nail breakage would be low. When the load increased, the von Mises stress increased in all three internal fixation techniques. The maximum von Mises stress values of Groups D, E, and F were 474.28, 436.31, and 413.4 MPa under an axial load of 750 N, respectively. The von Mises stress was consistent with the increase of the axial loads from 250 to 500 N, and Table 6 showed the maximum von Mises stress values of different loads. Moreover, the maximum von Mises stress in bone of Groups D, E, and F were 143.92, 72.73, and 67.29 MPa under an axial load of 750 N, respectively (Table 7). The maximum von Mises stress in bone by the novel plate was decreased compared to the posterior buttress plate and the lateral locking plate for PL depression fracture.

### 3.3 Anatomic Study

**FIGURE 8:**
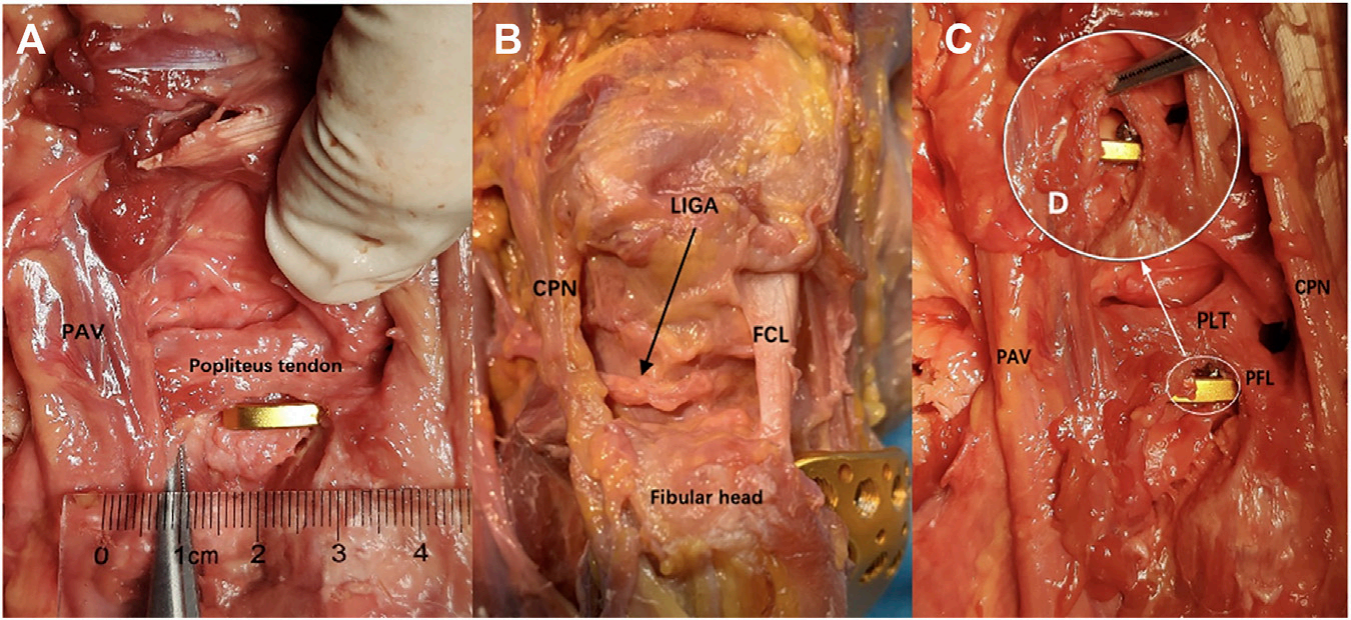
Anatomical study and fixation feasible. **(A)** Measurement of the distance between the tip of the “hoop hook” and PAV. **(B)** The relationship between the novel plate and key structures. **(C)** The relationship between the novel plate and the anterior wing screw. **(D)** Local enlarged view (PLT, the popliteus tendon; PAV, the popliteal arteriovenous vessels; CPN, the common peroneal nerve; LAGA, the lateral inferior genicular artery; FCL, the fibular collateral ligament; PFL, the popliteofibular ligament).

The “hoop hook” of the novel plate was easily fixed to the PL tibial plateau through the superior fibular head space. The common peroneal nerve was identified on the posterior border of the biceps femoris and coursed through the PL aspect of the knee. Figure 8A demonstrates that the “hoop hook” and the common peroneal nerve were not in the same plane, and the common peroneal nerve was less likely to be damaged. On the basis of measurement and observation, we found that the mean distance between the tip of the “hoop hook” and popliteal arteriovenous vessels was 10.3 mm (range: 6–15 mm); therefore, the risk of injury to popliteal arteriovenous vessels was considered very low (Figure 8A). We carefully dissected the lateral inferior genicular artery. We observed that the lateral inferior genicular artery originated from the popliteal artery, running deep to the lateral collateral ligament at the joint line (Figure 8B). Compared with the position of the novel plate, the position of the lateral inferior genicular artery was higher. The average distance from the novel plate to the lateral inferior genicular artery was 8.2 mm (range: 6–12 mm). Therefore, the risk of injury to the lateral inferior genicular artery was also considered low. Moreover, the average distance from the upper edge of the novel plate and the articular surface was 5.3 mm (range: 4–6.2 mm). Additionally, an anterior wing screw of appropriate length was less likely to damage the popliteus tendon (Figures 8C, D).

## 4 Discussion

An increasing number of studies have shown that good long-term outcomes of tibial plateau fracture are associated with anatomic articular reduction and stable fixation (20). However, it is particularly difficult to achieve anatomic reduction and rigid internal fixation in complex tibial plateau fractures, particularly those involving the PL column. The treatment of PTPFs has been one of current hotspots (Sun et al., 2018). Currently, there is no consensus about the optimal internal fixation selection for PTPFs treatment due to the complicated structures of the posterolateral corner of the knee including the fibular head, the fibular collateral ligament, the popliteus tendon, and the peroneal nerve, which impedes the exposure and fixation of the fracture fragments (Heidari et al., 2013; Giordano et al., 2020; Song et al., 2020). The PTPFs treatment was proposed by some experts to use a posterior buttress plate *via* various posterolateral approaches to expose the fracture fragment directly. (Lin et al., 2015) exposed the operation area, repaired the fracture under direct vision through a posterolateral inverted “L” shape approach, and fixed it with a posterior plate fixation. However, the anterior tibial artery perforates through the interosseous membrane is located approximately 4.6 cm below the joint line (Heidari et al., 2013), which means that the safe area for anatomy and the length of the plate is limited (Kfuri and Schatzker, 2018). Lobenhoffer introduced a fibular head osteotomy technique for the PTPFs treatment (Lobenhoffer et al., 1997). The fracture can be exposed fully and fixed with this approach, but it is inevitable to cause iatrogenic injuries on the posterolateral corner of the knee. On the other hand, many surgeons had successfully used lateral plate fixation to manage PTPFs *via* an extend anterolateral or anterolateral supra-fibular-head approach (Hu et al., 2016). Anterolateral operative approaches are relatively simple, and the risk of injury to the neighboring structures is low. Moreover, the lateral locking plate provided good support for depression fracture fragment (Jordan et al., 2016). Sun thought that lateral locking plate fixation was still one of the main trends for the treatment of PTPFs (Sun et al., 2018). However, compared with the posterior buttress plate, the lateral plate used in the PL split fracture fixation is still controversial owing to the weakness of the anti-shear effect (Zhang et al., 2012; Sun et al., 2018). So, there is a great need of PTPFs treatment concerning to low surgical risk, low iatrogenic injury, and rigid fixation.

Currently, studies on the treatment of PTPFs have mainly focused on surgical approaches, and operative fixation devices for the treatment of PTPFs have rarely been reported. The available types of internal fixation are relatively single and limited. In clinical practice, although the anterolateral approach is relatively simple and safe, with a short learning curve, failure cases with PTPFs treatment with the lateral plate fixation are often encountered (Wang et al., 2017). Thus, we designed a novel plate via the anterolateral approach, which could provide adequate support and rigid fixation. The novel plate has a “hoop hook,” which is behind the transverse arm of the L-shaped plate, parallel to the articular surface. The “hoop hook” hugs the PL split fragment, which can resist the sliding and posterior displacement of the fracture fragment. And the novel plate has an anterior wing in front of the longitudinal arm of the L-shaped plate and the locking screw into the screw hole of the anterior wing can mount on the “hoop hook,” thus combining the anterior wing screw and the “hoop hook” as a whole to fix the PL fragment. The “hoop hook” concept design is similar to the rim plate concept in recent years (Cho et al., 2016; Cho et al., 2017). Compared with the novel plate, the rim plate lacks a plate body that extends to the tibia shaft. Therefore, it is difficult for the rim plate to apply alone when the PL fragment is comminuted and depressed (Cho et al., 2016; Giordano et al., 2020). Meanwhile, the rim plate is usually obtained by pre-bending the distal radius plate, and a thicker plate increases the difficulty of pre-bending. When the depression fracture sometimes extends to the lateral column of tibial plateau, the rim plate is required to combine with other internal fixations, which means a longer operative time is required (Yi et al., 2020). The “hoop hook” of the novel plate is relatively thin, making adjustments relatively simple if necessary, and anatomical design of hook can match the morphological structure of most PL tibial plateau. The design concept of the anterior wing screw on the novel plate is similar to that of the magic screw (Sun et al., 2017; Sun et al., 2018). An additional screw can be placed, and this screw does not interfere with plate fixations when fixing the PL fragment. The strength of the magic screw fixation with the proximal tibial cortex was weaker than that of the combination fixation of screw and plate. Sun (Sun et al., 2018) proposed that the PTPF fragment would easily lose the reduction following the traditional process by screwing because the fragment was relatively small and had no supporting point. Low support of the magic screw might cause further displacement of the PL fragment of fracture. Interestingly, when the novel plate was used to fix the PL split fragment, we found that the posterior “hoop hook” provided the support for the fragment while hugging the fracture, and the anterior wing screw was placed in a defined thread direction to avoid interference with the proximal screws. Therefore, the anterior wing screw was easier to insert into the PL split fragment. A previous study (Zhang et al., 2012) showed that posterior buttress plating could provide the stronger fixation than lateral locking plate for PL split fracture. According to our biomechanical test, the novel plate showed better biomechanical strength for the PL split fracture than the posterior buttress plate and lateral locking plate. And the result of finite element analysis also showed that there was the least displacement of PL fragment in the novel plate group, and had the similar tendency with the biomechanical testing. Meanwhile we analyzed the maximum von Mises stress and stress distributions through three-dimensional (3D) computational model of the finite element. The novel plate exhibited a significantly lower maximum von Mises stress than the posterior buttress plate and lateral locking plate, although the stress concentration point appeared at the contact point of the “hoop hook” and the anterior wing screw. From another perspective, the stress concentration point indicated the “hoop hook” and the anterior wing screw played a role of resistance in the displacement of the PL fragment. Moreover, the maximum von Mises stress of the stress concentration observed in the “hoop hook” was relatively low compared to the yield strength of 800 MPa in the plate material at 300% body-weight loading; this suggested that no failure risk of mechanics would be expected in the implants.

Compared with PL split fracture, the PL depression fracture, which occurs more frequently in PTPFs, has been conspicuously overlooked. According to previous studies (Giordano et al., 2020; Yi et al., 2020), posterior buttress plating fixation was unsuitable for the PL depression fracture, and our biomechanical test also proved it. Compared with posterior buttress plating, the novel plate and lateral locking plate provided stronger biomechanical supports. This suggested that the strength of the novel plate and locking plate might be more suitable for pure PL depression fractures. Clinically, PL depression fracture of tibial plateau with posterior wall breakage is very common and bone graft is often needed to support the depressed articular surface. If the posterior wall is broken, bone graft would shift to the back side and ineffective bone graft without support to the articular surface would occur. For that matter, the novel plate with hoop hook, which can resist posterior wall displacement, was better than lateral locking plate for posterolateral depression fracture with posterior wall breakage. Furthermore, according to the finite element analysis, the novel plate had balanced stress distribution. And the maximum von Mises stress on the novel plate was relatively lower than lateral locking plate, which meant the risk of the novel plate failure was lower compared with lateral locking plate.

The novel plate was inserted through the lateral incision into the knee joint. This surgery had the advantage of being a low-risk, relatively simple operation. Moreover, the anatomic study showed that the “hoop hook” could be inserted through the superior fibular head space and was enough space from important structures such as major blood vessels and nerves. Therefore, the novel plate had the low risk of iatrogenic injury to the important structures of the PL corner of knee joint. In general, the novel plate showed a good biomechanical property either for posterolateral split fracture or for posterolateral depression fracture, which would have great clinical application.

There are several limitations to this study. First, instead of using human cadaveric bone, which was an ideal test material, we used synthetic tibia in our study. However, synthetic bone provided several advantages over human cadaveric bone. The synthetic tibiae provided standard dimensions and properties between specimens, and the geometrical measurements of the models were obtained from the same mold to ensure the uniformity of the experimental specimens. Second, the biomechanical evaluation in our study was relatively simple; the factors influencing knee stress and stability, including ligaments, muscles, and other soft tissues was not involved in this experiment. Third, the morphology of the PL depression fracture in clinic consists of two typical types: pure depression fracture and depression fracture with posterior plateau rim breakage, with the latter being more common. However, the modeling of the depression fracture with posterior plateau rim breakage is hard to simulate and construct. The effectiveness of the novel plate for depression fracture with plateau rim breakage had not been verified. Further designs of fracture modeling would be required. In addition, the morphology of the PL fracture was various and diverse in clinic, with two typical models, pure split fracture and pure depression fracture, hard to simulate all fracture morphology in clinic. Finally, the biomechanical result of this study must be interpreted as strictly static biomechanical testing, representing only part of the scenario at work and the state of a single-leg stance. The clinical application of the novel plate for PL fracture needed to be further verified.

## 5 Conclusion

The current study showed the novel plate had a good biomechanical advantage for PL tibial plateau fracture. And the finite element analysis suggested the novel plate had balanced stress distribution and low risk of fixation failure. Moreover, the placement of the novel plate had a low risk of damage to the important anatomic structures of the knee posterolateral corner through anterolateral approach. The novel plate may be a great choice for the treatment of PTPFs.

## Data Availability

The original contributions presented in the study are included in the article/Supplementary Material, further inquiries can be directed to the corresponding authors.
